# Dry-Cured Bísaro Ham: Differences in Physicochemical Characteristics, Fatty Acid Profile and Volatile Compounds Between Muscles

**DOI:** 10.3390/foods14142474

**Published:** 2025-07-15

**Authors:** Lia Vasconcelos, Luís G. Dias, Ana Leite, José M. Lorenzo, Alfredo Teixeira, Sandra S. Q. Rodrigues, Javier Mateo

**Affiliations:** 1 CIMO, LA SusTEC, Instituto Politécnico de Bragança, Campus de Santa Apolónia, 5300-253 Bragança, Portugal; lia.vasconcelos@ipb.pt (L.V.); ldias@ipb.pt (L.G.D.); anaisabel.leite@ipb.pt (A.L.); teixeira@ipb.pt (A.T.); srodrigues@ipb.pt (S.S.Q.R.); 2 Department of Food Hygiene and Technology, University of León, Campus Vegazana S/N, 24007 León, Spain; 3 Centro Tecnológico de la Carne de Galicia, Avd. Galicia N 4, Parque Tecnológico de Galicia, San Cibrao das Viñas, 32900 Ourense, Spain; jmlorenzo@ceteca.net

**Keywords:** physicochemical characteristics, fatty acid profile, volatile compounds, Bísaro breed, dry-cured ham, muscle type

## Abstract

The aim of this study was to evaluate differences in the physicochemical characteristics, fatty acid profiles and volatile compounds of different muscle types (*semimembranosus* (SM), *biceps femoris* (BF) and *semitendinosus* (ST)) used to produce dry-cured Bísaro ham. Sixteen dry-cured hams were used. The physicochemical parameters were significantly affected by the muscle type, with the differences being mainly related to the different drying degrees and the intramuscular fat and collagen contents of the fresh muscles. Additionally, the type of muscle had a significant influence on the polyunsaturated fatty acids, such that the muscle with the highest fat content (ST) had the lowest PUFA content and vice versa. There were strong significant differences in the total content of volatile compounds derived from the Strecker reaction, which was higher in the ST muscle, and in the proportions of these compounds with different functional groups. The amount of sulfur compounds was also affected by the muscle type and was higher in the SM muscle. Due to the great impact of Strecker-derived and sulfur compounds on the flavor of the cured hams, these differences would affect the flavor perception of the different muscles. The variability between muscles in composition, fatty acids and volatile compounds allowed for discrimination of the samples by muscle type using multivariate analysis.

## 1. Introduction

The Bísaro is a pig breed of Celtic origin from the north of Portugal, particularly from the Trás-os-Montes region. This breed adapts well to traditional management and extensive and semi-extensive farming regimes, where feeding is based on commercial feeds supplemented with other local feeds of vegetable origin, such as chestnuts, turnips, potatoes, pumpkins, beets, fruits and green cereals. Pork and pork products from pigs of this breed are highly appreciated by consumers [1]. Under European Union regulations [2], Bísaro pork can be categorized under several certified products (protected designation of origin (PDO) and protected geographical indication (PGI)), ranging from ‘Carne de Bísaro Transmontano’ fresh meat to ‘Presunto Bísaro de Vinhais’ dry-cured hams, which are the subject of this research.

Similar to a wide variety of meat products around the world, the production and consumption of dry-cured ham in Portugal has been gaining ground [3,4,5,6,7,8]. In the Iberian Peninsula, there is a wide variety of dry-cured hams that are differentiated based on the characteristics of the raw meat (genetic type, feed, breeding system, slaughter weight, meat/fat ratio, etc.), the use of non-meat ingredients (amount of salt, spices, seasonings, etc.) and the ripening conditions (time, temperature and relative humidity) [4,9,10].

Moreover, from an anatomical point of view, the moisture and fat contents, fatty acid composition [11], moisture loss and a variety of biochemical changes that take place in the ham during ripening can be affected by the type of muscle [9,12] or by the trimming method used, e.g., removing or not removing part of the skin and subcutaneous fat [13]. Among the muscles that make up dry-cured hams, three muscles which constitute most of the central part of the ham can be easily distinguished, namely *biceps femoris* (BF) and *semitendinosus* (ST), which are both internal muscles covered with skin and a thick layer of fat, and *semimembranosus* (SM), an external muscle without a skin or fat cover and thus subject to more rapid salt absorption and drying [9]. The effects of muscle-related variables on dry-cured ham quality are mediated by the complex reactions that take place during ripening and are mostly due to water loss, salt intake and lipolytic, proteolytic and oxidative processes [4]. Specifically, depending on the anatomical localization (muscle), different degrees of proteolysis and lipolysis can be expected, with greater activity in the muscles of the interior, which could affect the quality traits including flavor and texture [14,15,16].

Free fatty acids, amino acids and peptides derived from lipolytic and proteolytic processes serve as the substrates for lipid oxidation and the Strecker reaction. These reactions produce a large variety of volatile compounds (VOCs), such as organic acids, alcohols, aldehydes, ketones, esters, lactones, furans and hydrocarbons, some of which have distinct aromatic notes that are responsible for the flavor of dry-cured hams [17]. At the industrial level, these flavor compounds have a significant impact on the characterization and valorization of the final product [8].

The objective of this study was to investigate the relationship between the muscle type and quality traits of Bísaro dry-cured ham by studying the effect of muscle type on the physicochemical characteristics and fatty acid and volatile compound profiles. The general purpose was to provide useful information to aid in improving the production process for this type of dry-cured ham.

## 2. Materials and Methods

### 2.1. Dry-Cured Bísaro Ham Production Process

Dry-cured Bísaro hams were prepared from purebred Bísaro pigs reared in a commercial farm in north-eastern Portugal (Bísaro Salsicharia Tradicional; Gimonde, Bragança, Portugal). On this farm, the pigs were raised under a semi-extensive production system according to regional livestock practices, with their diet supplemented with commercial feed and seasonal vegetables. This system promotes the welfare of the animals by giving them space to explore and graze. As in any licensed commercial farm, during rearing, the animals were cared for in accordance with welfare standards and in compliance with EU Council Regulation (EC) No. 1099/2009 [18]. For the experiment, 16 pigs were purchased, all 12 months of age and weighing approximately 135 kg. These 16 animals were randomly selected from 40 pigs on the farm because they met these conditions, resulting in 10 castrated males and 6 females for the study. The animals were slaughtered in an authorized slaughterhouse following the slaughtering and carcass preparation procedures previously described by Álvarez-Rodríguez and Teixeira [19]. The mean carcass weight was 110 kg.

After chilling the carcass for 24 h at 0–4 °C, a main leg was removed from each carcass. Each leg was then trimmed by cutting away some of the lean meat, fat and skin to obtain the typical ham shape [20]. The hams were left to rest (for 24 h under refrigeration) and then compressed with a hand massage to allow the internal liquids—mostly blood retained in large blood vessels—to be properly eliminated. Afterwards, the hams were subjected to salting and dry ripening at the Bísaro Salsicharia Tradicional meat products processing plant according to a traditional method for a total of 16 months. Briefly, salting was carried out by covering the legs with salt, which were then left to stand for a time depending on the weight of the pieces (1 day per kg of weight). This phase took place in a static refrigeration chamber at a temperature of 0–2 °C and a relative humidity (RH) of 80–95%. No curing agents (nitrites or nitrates) were added during this stage, according to the protected geographical indication for Bísaro ham regulation. After salting, the salt adhered on the ham surface was removed using pressurized warm water, and the legs were then hung in a chamber (0–3 °C, 85–90% RH) for the 3-month post-salting stage.

The hams were then moved to a ripening chamber (first drying step) where the temperature was gradually increased from 3 °C to 8 °C and the RH was dropped to 80–85% for a period of 4 months. Afterwards, the hams were transferred to another ripening chamber (second drying stage) where the temperature was gradually increased from 8 °C to 16 °C and the RH was reduced to 75–80% for another 4 months. Finally, the hams were kept for 9 months in a third ripening chamber (final ripening stage) where the RH was set at 60–70% and the temperature was gradually increased from 16 to 30 °C for the first 4 months and then lowered to 18–20 °C. Once dry-cured, the hams were taken from the plant to the Carcass and Meat Quality Laboratory at the School of Agriculture of the Polytechnic Institute of Bragança (Bragança, Portugal) for sampling and analysis.

### 2.2. Dry-Cured Bísaro Ham Muscle Sampling

**Figure 1:**
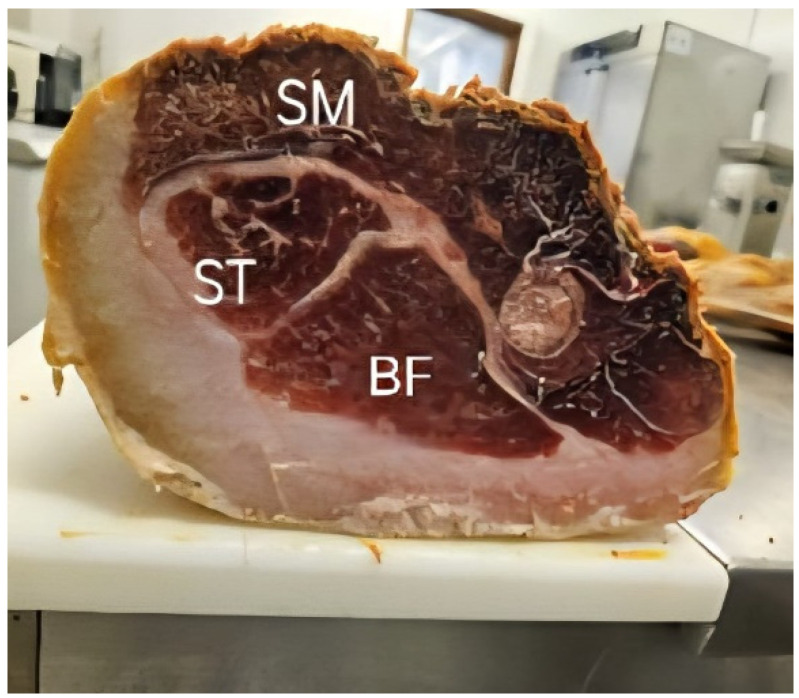
Image of the three muscles *semimembranosus* (SM), *biceps femoris* (BF) and *semitendinosus* (ST) in a dry-cured Bísaro ham.

Once in the laboratory, the hams were skinned and deboned, and the SM, BF and ST muscles (Figure 1) were dissected and used for the analyses of water activity (a_w_), NaCl content, proximate composition, collagen and heme pigment concentrations and fatty acid and volatile compound profiles (Figure 1). The pH was measured using a Crison 570 pH meter equipped with a 52-32 puncture electrode (Crison Instruments, Barcelona, Spain) following Portuguese standard NP-ISO 3441/2008 [21]. The whole portion of each muscle was then minced using a Buchi Mixer B-400 power mill (BÜCHI, Labortechnik AG, Postfach, Flawil, Switzerland) for 10 s until a homogeneous paste was obtained, from which about 100 g was taken for the analyses.

### 2.3. Proximate Composition, Sodium Chloride, Collagen, Heme Pigment Contents and a_w_

Moisture was determined according to the Portuguese standard [22] by drying the sample in an oven (Raypa DO-150, Barcelona, Spain) for 4 h at 103 ± 2 °C. The ashes were assessed according to the Portuguese standard [23] using a muffle furnace (Vulcan BOX Furnace Model 3-550, Yucaipa, CA, USA). Fat (intramuscular fat) was extracted from a 25 g muscle sample according to the Folch procedure [24] and weighed. The protein determination was carried out following the Portuguese standard [25] using the Kjeldahl Sampler System (K370, Flawil, Switzerland) and Digest System (K-437, Flawil, Switzerland). The total chloride content was quantified according to Portuguese standard NP 1845 [26] and calculated as the NaCl content. The collagen content was quantified according to Portuguese standard 1987 [27]. The total heme pigment content was determined according to Hornsey’s procedure [28] and the absorbance at 512 nm was measured using a Spectronic Unicam 20 Genesys spectrophotometer (SPECTRONIC 20 GENESYS, Thermofisher Scientific, Austin, TX, USA) and expressed as mg myoglobin/g sample. The water activity (a_w_) was assessed according to AOAC [29] using a HigroPalmAw1 Rotronic 8303 probe (Bassersdorf, Switzerland). Three repetitions were carried out for each variable.

### 2.4. Fatty Acid Composition Analysis

From the fat extracted using the Folch method [24], a 50 μg sample, in duplicate, was used to analyze the fatty acid profile of the dry-cured ham muscles. The fatty acids were transesterifed following the method of Domínguez et al. [30], and the fatty acid methyl esters were analyzed using chromatography following the procedure reported by Teixeira et al. [31]. Fatty acid methyl ester separation was performed using a gas chromatograph (GC-Shimadzu 2010Plus; Shimadzu Corporation, Kyoto, Japan) equipped with a flame ionization detector and an AOC-20i automatic sample injector and using a Supelco SP-2560 (Bellefonte, PA, USA) fused silica capillary column (100 m length, 0.25 mm internal diameter, 0.2 µm film thickness; Supelco). Identification was carried out using the standard Supelco 37 Component FAME Mix (Supelco). The fatty acid contents, expressed as g per 100 g of total fatty acid methyl esters, were calculated based on the chromatogram peak areas. To analyze the healthiness of the fatty acid profile of the dry-cured Bísaro ham muscles, the polyunsaturated (PUFA)/saturated (SFA) fatty acid ratio, the atherogenicity (IA) and thrombogenicity (IT) indices proposed by Ulbricht and Southgate [32] and the hypocholesterolemic/hypercholesterolemic (h/H) ratio [3] were calculated.

### 2.5. Volatile Compound Profile

One gram of the homogenized SM, BF and ST muscles was transferred to 10 mL screw cap headspace vials. The volatile compound profile was obtained by solid-phase micro-extraction (SPME) coupled to gas chromatography–mass spectrometry using an Agilent Technologies (Santa Clara, CA, USA) chromatograph equipped with a DB-624 capillary column (30 m length, 0.25 mm internal diameter, 1.4 μm film thickness; J&W Scientific, Folsom, CA, USA) and a mass-selective detector 5977B MSD (Agilent Technologies) following the procedure described by Dominguez et al. [4]. The linear retention indices (LRIs) of the volatile compounds were calculated. The identification of volatile compounds was carried out based on matching the peak spectra both with a spectral database and the relative retention time. The results are expressed as the area units per gram of sample (10^5^ AU/g of the sample). The determination of these compounds was performed at Centro Tecnologico da Carne (CTC) (San Cibrao das Viñas, Ourense, Spain).

### 2.6. Statistical Analysis

Data on the physicochemical composition and fatty acid and volatile compound profiles were analyzed using the general linear model (GLM) procedure. The quality characteristics were defined as dependent variables, and muscle was considered the fixed effect, with ham as the experimental unit. The results are presented as the mean values with the standard error of the mean (SEM). When a significance level of *p* < 0.05, *p* < 0.01 or *p* < 0.001 was detected, Tukey’s HSD test was used to compare the means. All analyses were conducted using the statistical software JMP^®^ Pro 16.0.0, 2021 edition (SAS Institute Inc.©, Cary, NC, USA). Principal component analysis (PCA), an unsupervised multivariate method, and linear discriminant analysis (LDA), a supervised classification technique, were applied to the datasets to analyze the physicochemical parameters and fatty acid and volatile compound profiles. Prior to the multivariate analysis, all variables were mean-centered and scaled to unit variance to ensure equal importance in the mathematical treatment and to prevent biases arising from differences in the variable magnitudes.

## 3. Results and Discussion

### 3.1. Proximate Composition, Sodium Chloride, Collagen and Heme Pigment Contents, a_w_ and pH

Table 1 shows the results for the different physicochemical characteristics of the dry-cured Bísaro ham muscles. Significant differences were observed between the muscles for all the characteristics assessed (*p* < 0.01 or *p* < 0.001) except for pH, which had an average value of 5.70 for the three muscles.

The BF muscle showed the highest a_w_, which was significantly different from that of the SM muscle. The BF muscle also contained the highest moisture content, with significant differences compared to the other two muscles. The BF and ST muscles had similar moisture/protein ratios (1.7 vs. 1.5, respectively); however, the SM muscle showed a lower value (1.0). The observed differences in moisture content and a_w_ between the BF and SM muscles can be mainly attributed to the greater degree of drying of the SM muscle which, unlike the BF muscle, is exposed to the ham surface and thus exposed to evaporative losses. The results are in accordance with those found in Iberian [10], dry-cured Celta [9,33] and Slovenian hams [12]. Water loss is the most influential change in dry-cured hams [33]. Consequently, due to the high moisture loss, the SM muscle exhibited the highest protein and collagen contents (*p* < 0.001).

The ST muscle of the dry-cured Bísaro ham had the highest intramuscular fat content when fresh (and dried). The origin of this difference lies in the degree of marbling of the fresh muscle. Different results were observed in other types of ham [12,15] with a higher fat content when comparing SM to BF.

The ash and NaCl contents were significantly higher in the BF muscle compared to in SM and ST (*p* < 0.01). This difference is likely related to the differences in the moisture and fat contents between the muscle types rather than a non-homogeneous distribution of the salt used in the curing process, as the salt/moisture ratios for the three muscles were similar (approximately 14 g NaCl/100 g H_2_O).

Regarding the color-related parameters, the SM muscle, the driest one, contained the highest heme pigment content (2.60 mg/g sample), significantly surpassing that of BF and ST (*p* < 0.001). This difference might contribute to the darker color of this muscle [28]. The negative relationship between the moisture and heme pigment contents was weak due to differences in the heme pigment/protein ratios between the muscles (6.3, 5.2 and 6.2 mg heme pigments/g protein for ST, BF and SM, respectively). This means that there was a difference in the heme pigment contents between the muscles before drying. Likewise, the differences in collagen content depend not only on the degree of drying [34] but also on differences in the collagen content of the fresh muscles. When the collagen content is calculated as a percentage of the total protein content and is therefore independent of moisture, the collagen contents of ST, BF and SM were 6, 5 and 4%, respectively. These results highlight the muscle-specific variability in dry-cured ham, emphasizing the need to consider anatomical differences when evaluating product quality.

### 3.2. Fatty Acid Composition

The fatty acid profile of the intramuscular fat of the dry-cured ham muscles is shown in Table 2. For brevity, only the most abundant fatty acids (with a proportion higher than 2% of the total fatty acids) were included in the table. Numerous factors, such as the breed, sex, rearing and feeding systems, slaughter weight, age, muscle type and curing time, can affect the fatty acid profile of dry-cured ham muscles [35,36]. Despite these variables, oleic acid is typically the predominant FA in the intramuscular fat fatty acid profile of this type of pork product, followed by palmitic and stearic acids. This pattern has been reported in dry-cured Bísaro loin and cachaço [1], Iberian dry-cured shoulder [7] and hams of other origins [3,35].

There were significant differences in the percentages of PUFAs between the muscles. The SM muscle had the highest PUFA content, followed by the BF and ST muscles. Consequently, among individual PUFAs, the percentage of linoleic acid (C18:2n-6), which was the major component, was significantly higher in the SM muscle, followed by the BF muscle and then the ST muscle (*p* < 0.001). In contrast, neither the total SFA or MUFA content differed significantly between the muscles.

The fatty acid profiles found in this study were consistent with the profiles reported for dry-cured hams from other traditional pig breeds [9,11]. The PUFA differences can be largely explained by the different intramuscular fat contents between the muscles. The higher the intramuscular fat content, the lower the degree of polyunsaturation due to the greater presence of triglycerides in the marbling fat as opposed to phospholipids, which are abundant in membrane fat. PUFA variability might affect the oxidative stability, flavor development and nutritional value of the ham.

The differences in PUFAs were not large enough to result in significant differences in the IA, IT and h/H ratio between the muscles. No organization has provided recommended IA and IT values [37], but the IA (~0.44–0.46) and IT (~0.99–1.07) values obtained in this study are in line with those reported for traditional cured hams. However, the PUFA/SFA ratio differed significantly between the muscles, similarly to the differences in PUFAs. Even so, these ratios are below those recommended by the Department of Health and Social Care [38] (0.4). In summary, the higher PUFA content in the SM, coupled with a lower n-6/n-3 ratio, suggests a lipid profile with greater nutritional value but potentially increased susceptibility to oxidative degradation. Conversely, the ST muscle, with a lower PUFA content, may exhibit improved oxidative stability but a less favorable nutritional profile regarding essential fatty acids.

### 3.3. Volatile Compounds

A total of forty-nine compounds were quantified in the samples from the SM, BF and ST muscles (Table 3). These compounds were classified into six chemical families: esters (12), hydrocarbons (11), alcohol (7), ketones (7), aldehydes (5) and other compounds (7), mostly sulfur compounds.

The higher number of compounds found in the dry-cured hams were mainly derived from lipid oxidation, i.e., straight-chain aldehydes, alcohols, acids and esters; most of the hydrocarbon; ketones (except acetoin); and 2-pentylfuran [39,40]. Nonetheless, the overall concentration of these compounds, particularly straight-chain aldehydes such as hexanal, which are closely associated with lipid oxidation in dry-cured hams [17,41], was comparatively low. Their levels were notably lower than those of more abundant volatile compounds, including 3-methylbutanol, 3-methylbutanal and toluene, which originate from alternative biochemical pathways. This suggests that the lipid oxidation in the ripening of the dry-cured hams was a controlled process and that the higher proportion of PUFAs in the SM muscle did not result in a higher lipid oxidation rate. In this context, it has even been observed that aldehydes tend to decrease during the ripening of dry-cured hams [9]. At high concentrations, hexanal is strongly involved in the development of rancid aromas, while at moderate levels, such as those found in the hams in this study and others [12,42,43], hexanal seems to contribute to the characteristic odor of the meat product [4,41,44]. Aliphatic hydrocarbons were the most abundant lipid-derived volatile compounds in the dry-cured hams due to the high levels of octane, 2,2,4,4–tetramethyloctane and heptane. The high abundance of these latter two compounds in dry-cured hams has also been described by other authors, but they were not thought to have an important role in the flavor of the ham [41,43,45,46,47].

The second largest group of volatile compounds in the dry-cured hams and the most abundant in concentration consisted of branched-chain and aromatic aldehydes, alcohols and esters. These compounds may have come from the deamination of amino acids, i.e., the Strecker reaction. They have been found to be abundant in dry-cured hams and are considered very important for achieving their full flavor [4,35,45,46].

Among the compounds derived from lipid oxidation and Strecker degradation in the dry-cured ham, the presence and formation of aldehydes, alcohols and esters seemed to be related to each other, and their quantities and distributions were highly dependent on the ripening time. During ripening, alcohols are formed through the reduction of aldehydes, and esters are formed by the esterification of alcohols with organic acids [48]. Both alcohols and esters seem to increase during the last months of ripening [12]. Thus, the hams in this study were expected to have high alcohol and ester contents due to the long maturation period of 16 months.

A third less abundant but characteristic group of volatile compounds in the dry-cured hams comprised sulfur compounds, presumably originating from the catabolism of sulfur-containing amino acids [39]. Sulfur compounds have very low odor threshold values [40] and, according to Martínez-Onandi et al. (2017) [10], dimethyl disulfide is highly implicated in the flavor of dry-cured hams, contributing notes of cabbage, garlic and onions.

Regarding the different muscles, there were no clear differences in the amounts of lipid-derived aldehydes or ketones since there were no significant differences between the most representative and abundant ones (hexanal, nonanal, 2-butanone, 2-pentanone and 2-nonanone); all of these compounds have a low odor threshold [49]. These results partially agree with those of Plugiese et al. [12], who found no difference in hexanal and 2-heptanone levels between BF and SM muscles but did find higher levels of nonanal in BF muscle.

There were higher levels of straight-chain alcohols (2-butanol, 1-pentanol and 1-hexanol) in the ST muscle and lower levels of 1- and 2-pentanol and 1-hexanol in the SM muscle, suggesting higher and lower levels of conversion of alcohols from aldehydes in these muscles. In a study on Kraški pršut ham, higher levels of straight medium-chain alcohols like hexanol were also found in SM compared to in BF muscle [12]. The concentration of medium (from 4 to 9 carbons)-length straight-chain alcohols was significantly lower in the SM muscle when compared with that in the other muscles. The differences in the abovementioned alcohols between the muscles would have a small effect on flavor, due to their relatively high levels [50]. Lorenzo et al. [45] have suggested that alcohols contribute herbaceous, woody and fatty notes to dry-cured products.

There were more significant differences in the compounds derived from Strecker degradation between the muscles. The levels of Strecker reaction-derived aldehydes, i.e., 3-methyl butanal, benzaldehyde and benzene acetaldehyde, were higher in the BF muscle. Meanwhile, those with alcohol and ester functional groups, i.e., 3-methylbutanol, benzyl alcohol and 2- and 3-methylbutyl acetate, were more abundant in the ST muscle than in the two other muscles. Overall, the ST muscle had the highest number of products from the Strecker degradation of amino acids (240 × 10^5^ AU/g), with BF having the lowest amount (200 × 10^5^ AU/g).

Due to the difference in the total amount of compounds from the Strecker reaction and the fact that alcohols and esters form gradually from aldehydes through chemical reduction, there may be a lower rate of degradation of amino acids and lower reduction rates in the BF muscle than in the ST muscle, with intermediate rates in the SM muscle. The differences in the levels of these types of compounds may have an important effect on the flavor of the muscles. Aldehydes and alcohols from the degradation of amino acids have a low olfactory threshold and are responsible for the typical aroma of dry-cured hams [4,12,45,46,51,52]. Moreover, esters from short-chain acids would result in the generation of fruity notes, whereas the formation of esters from long-chain acids would give rise to fatty odor notes [12,17].

Sulfur compounds clearly differed between the muscles, with the SM muscle showing the highest amounts of the three sulfur compounds detected (methanethiol, dimethyl disulfide and trisulfide). No differences were observed between the BF and ST muscles. Differences in sulfur compounds could imply flavor differences since they are important aroma-active compounds that contribute to the overall flavor of dry-cured hams [40]. Finally, the concentration of butanoic acid, which is associated with cheese and rancid odors, was higher in the BF muscle. Pluguiese et al. [12] also found more butanoic acid in BF muscle than in SM.

### 3.4. Muscle Profiles from Principal Component and Discriminant Analyses

**Figure 2:**
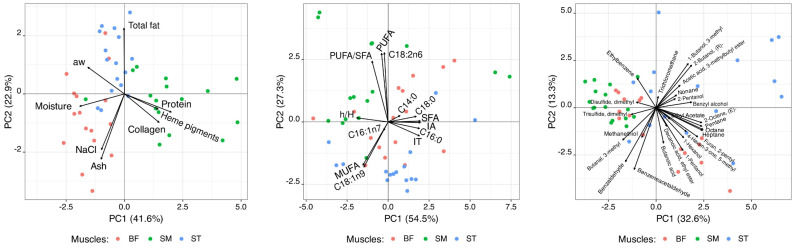
Biplots of principal component analysis (PCA) of *biceps femoris* (BF) (red), *semimembranosus* (SM) (green) and *semitendinosus* (ST) (blue) muscles based on physicochemical characteristics and chemical composition, as well as fatty acid and volatile compound profiles.

**Figure 3:**
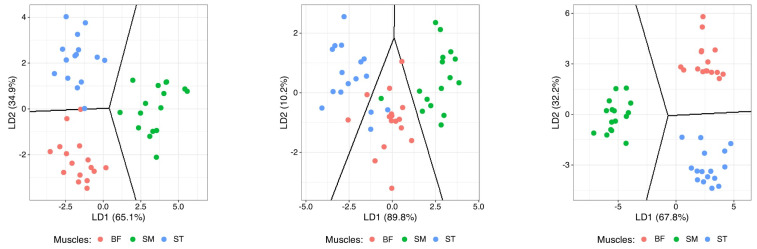
Biplots of linear component analysis (LDA) of *biceps femoris* (BF) (red), *semimembranosus* (SM) (green) and *semitendinosus* (ST) (blue) muscles based on physicochemical characteristics and chemical composition, as well as fatty acid and volatile compound profiles.

Figure 2 and Figure 3 show the principal component analysis (PCA) and linear discriminant analysis (LDA) results for the different types of muscle (SM, BF and ST) using the parameters with statistically significant differences (except for the fatty acid composition, where all the principal fatty acids were included).

From the PCA for the physicochemical characteristics and chemical composition, the first principal component (PC1) explained 41.6% of the variance, while the second component (PC2) explained 22.9%, totaling 64.5% of the variance (Figure 2). The positive region of the first factor (PC1) was mainly represented by the SM muscle while the negative region contained the BF and ST muscles. On the other hand, the positive region of the second factor (PC2) was mainly represented by the SM muscle while the BF muscle was in the negative region. This supports the results obtained in the physicochemical analysis where the BF and ST muscles were found to contain a higher a_w_ and moisture content due to their more internal muscle position. As for the collagen, protein and heme pigment contents, the multivariate analysis showed difference between the SM muscle and the other two muscles.

Using the covariance matrix without normalizing the variables, it was found that the most important variables were the protein, fat and moisture contents. The results of the linear discriminant analysis (LDA) had some limitations and challenges in their interpretation (Figure 3). The first linear discriminator (LD1) explained 65.1% of the variance, while the second (LD2) explained only 34.9%, indicating that there may not be a clear separation of the groups. The coefficients associated with the linear discriminants indicated that the variable *aw* had a markedly high weight in LD1 and LD2, while the collagen content stood out in LD2. The remaining variables contributed substantially less, raising questions about their relevance for group discrimination. The sensitivity and specificity for the SM muscle were 100% but there was a lower sensitivity (93.75) for the BF and specificity (96.88) for the ST, which indicates that the model had difficulty correctly differentiating between the different muscle types despite the high (97.92%) overall accuracy (confusion matrix.

The same behavior was observed with the fatty acid profile (Figure 2), leading to the conclusion that the lipid profiles differed between the different types of muscle. The results of the PCA showed that PC1 explained 54.5% of the variance, while PC2 explained 27.3%, totaling 81.8% of the variance, but some variables showed a large degree of dispersion and variability, complicating the situation (Figure 2). The SM muscle was again found to significantly differ from the other muscles (*p* < 0.001, Table 2), namely in terms of the C18:2n-6 and PUFA contents. These fatty acids could be used to differentiate the SM muscle from the other muscles. Using the variables and fatty acids that contributed most to differentiating between the muscles, another LDA was performed, which showed that the three muscles have different profiles (LD1 and LD2 explained 89.8% and 10.2% of the variance, totaling 100% of the variance) but complete differentiation of all the muscle samples was not achieved. The SFA, MUFA and PUFA contents, along with the h/H ratio, also exhibited considerable importance in both discriminant axes, particularly in LD2. In contrast, the content of individual fatty acids such as C16:1n7, C18:0 and C18:1n9 showed minimal contributions, indicating lower relevance for discriminating between the groups.

Finally, for volatile compounds, PC1 and PC2 explained 45.9% (32.6 and 13.3%, respectively) of the variation between samples (Figure 2). Although the PCA results showed good separation of the samples according to the type of muscle, a discriminant analysis was also performed. The statistical program selected twenty-five volatile compounds that contributed to the differentiation between the muscles. LD1 and LD2 explained 67.8% and 32.2% of the variance, totaling 100% of the variance. The coefficients associated with the linear discriminants revealed that the methanethiol content had by far the highest weight in LD1, while the 4-hexen-3-one,5-methyl and 2-octene, (E)^−^ contents were the most influential in LD2. Although some other compounds such as ethylbenzene, nonane and trisulfide, dimethyl also showed high contributions, most of the volatile compounds exhibited a relatively low discriminant power, suggesting limited relevance for group separation. In this sense, the sensitivity and specificity for all muscles were 100%, which indicates that the model correctly identified and separated the muscles (100% accuracy from confusion matrix. We can observe that the SM muscle differed from the other muscles in terms of linear alkane content (lower area in Figure 3).

## 4. Conclusions

This study represents the first comprehensive assessment of the different muscle types—*semimembranosus* (SM), *biceps femoris* (BF) and *semitendinosus* (ST)—in Bísaro dry-cured ham, regarding their physicochemical characteristics and fatty acid and volatile compound profiles after 16 months of ripening. The results showed that muscle type had significant effects on these characteristics. The semimembranosus muscle, which was exposed to the most evaporation drying, tended to have a lower moisture content, more protein and therefore a higher protein/moisture ratio. The concentration of intramuscular fat in the muscles, which was higher in the semitendinosus muscle, could be explained by the amount of intramuscular fat in the fresh muscles and by the degree of drying. Despite the higher amount of salt and ash in the BF muscle, the concentrations of these components (expressed relative to the moisture content) did not differ between the different types of muscle, indicating a homogeneous distribution of salt in the ham water. The main difference in the fatty acid profiles between the muscles was in the percentage of PUFAs, which was mainly due to the variability in intramuscular fat between the fresh muscles, as the PUFA percentage was inversely proportional to the fat content in the dry muscle. There were differences between the dry-cured ham muscles in terms of the contents of key aromatic compounds with low perception thresholds, which could affect their flavor in different ways. The ST muscle showed the highest total content of Strecker degradation compounds, but the distribution of these compounds varied between the muscles, with the proportion of aldehydes being more abundant in the BF muscle while alcohols were more abundant in the ST muscle. Moreover, the SM muscle showed the highest amount of sulfur compounds, and the BF muscle had the highest amount of butanoic acid.

The variability between the muscles, in terms of chemical composition and fatty acid and volatile compound profiles, was reasonably identified through the first two components in the principal component analysis. Moreover, discriminant analysis of the dry-cured ham composition characteristics and the volatile compound profile showed a good performance in discriminating samples by muscle type. Further research should focus on the biochemical and textural changes in the BF, SM and ST muscles, as well as their correlation with sensory attributes, using advanced detection methods such as electronic nose technology to gain deeper insights into the effects of the maturation process on Bísaro ham quality.

## Figures and Tables

**Table 1:** Physicochemical characteristics and chemical composition of *semimembranosus*, *biceps femoris* and *semitendinosus* muscles of dry-cured Bísaro ham (n = 16).

Parameter	Muscle			SEM	SIG
	SM	BF	ST		
a_w_	0.84 ^b^	0.86 ^a^	0.85 ^ab^	0.004	**
Moisture (%)	42.09 ^b^	49.30 ^a^	45.25 ^b^	0.953	***
Total Fat (%)	7.09 ^b^	6.89 ^b^	12.78 ^a^	0.563	***
Protein (%)	41.00 ^a^	29.65 ^b^	31.06 ^b^	0.774	***
Ash (%)	6.53 ^b^	7.43 ^a^	6.82 ^b^	0.164	**
NaCl (%)	5.86 ^b^	7.10 ^a^	6.17 ^b^	0.220	**
Heme Pigment (mg/g)	2.60 ^a^	1.57 ^b^	1.92 ^b^	0.142	***
Collagen (%)	1.79 ^a^	1.31 ^b^	1.10 ^c^	0.089	***

SIG—significance; SEM—standard error of the mean; a_w_—water activity; SM—*semimembranosus*; BF—*biceps femoris*; ST—*semitendinosus*. Heme pigment content is given as mg myoglobin/g sample. Different lowercase letters (a, b, c) in the same column indicate significant differences between muscles. ANOVA followed by a post hoc Tukey test. ** (*p* < 0.01); *** (*p* < 0.001).

**Table 2:** Fatty acid composition of *semimembranosus*, *biceps femoris* and *semitendinosus* muscles in dry-cured Bísaro ham (n = 16).

FA (%)	Muscle			SEM	SIG
	SM	BF	ST		
C16:0	24.20	24.76	24.91	0.293	ns
C16:1n-7	3.15	3.03	2.97	0.069	ns
C18:0	9.94	10.68	10.45	0.342	ns
C18:1n-9	50.08	50.30	51.81	0.620	ns
C18:2n-6	7.80 ^a^	6.84 ^b^	5.67 ^c^	0.205	***
SFAs	35.68	36.90	35.26	1.057	ns
MUFAs	54.10	54.27	57.37	1.106	ns
PUFAs	10.21 ^a^	8.83 ^b^	7.37 ^c^	0.283	***
PUFA/SFA ratio	0.28 ^a^	0.24 ^b^	0.21 ^c^	0.266	***
IA	0.44	0.46	0.44	0.018	ns
IT	0.99	1.07	1.04	0.041	ns
h/H	2.36	2.26	2.25	1.897	ns

ns—not significant; SEM—standard error of the mean; FA—fatty acid; SIG—significance; SM—*semimembranosus*; BF—*biceps femoris*; ST—*semitendinosus*; SFA—saturated fatty acid; MUFA—monounsaturated fatty acid; PUFA—polyunsaturated fatty acid; PUFA/SFA—polyunsaturated/saturated fat ratio; IA—atherogenicity index; IT—thrombogenicity index; h/H—hypocholesterolemic/hypercholesterolemic index. Only fatty acids with a proportion greater than 2% are presented in the table, but all detected fatty acids were used to calculate the totals and the indices. Different lowercase letters (a, b, c) in the same column indicate significant differences between muscles (*p* < 0.05). ANOVA followed by a post hoc Tukey test. *** (*p* < 0.001).

**Table 3:** Volatile compound contents (10^5^ area units/g of sample) of dry-cured Bísaro ham according to muscle type (n = 16).

VOCs		Muscle			SEM	SIG
	M/Z	BF	SM	ST		
2-Butanol, (R)-	45	0.87 ^b^	0.77 ^b^	2.37 ^a^	0.332	0.002
1-Butanol	56	1.12	0.82	1.33	0.187	0.175
2-Pentanol	45	14.21 ^a^	8.73 ^b^	12.85 ^ab^	1.541	0.041
1-Pentanol	70	7.58 ^ab^	5.78 ^b^	9.03 ^a^	0.872	0.039
1-Hexanol	69	16.96 ^ab^	12.54 ^b^	23.69 ^a^	2.543	0.012
1-Butanol, 3-methyl-	70	57.52 ^b^	81.80 ^b^	144.25 ^a^	14.320	<0.001
Benzyl alcohol (aromatic)	79	6.20 ^b^	4.33 ^c^	8.53 ^a^	0.540	<0.001
TOTAL ALCOHOL CONTENT		104.45 ^b^	114.77 ^b^	202.05 ^a^	15.605	<0.001
Butanal, 3-methyl-	58	51.20 ^a^	65.05 ^a^	19.26 ^b^	6.107	<0.001
Hexanal	56	27.48	20.59	12.68	4.383	0.068
Benzaldehyde (aromatic)	106	30.67 ^a^	31.09 ^a^	17.02 ^b^	2.473	<0.001
Benzeneacetaldehyde (aromatic)	91	32.62 ^a^	32.60 ^a^	19.27 ^b^	2.912	0.002
Nonanal	57	6.82	6.29	5.20	0.873	0.416
TOTAL ALDEHYDE CONTENT		148.79 ^a^	155.61 ^a^	73.43 ^b^	13.195	<0.001
Pentane	72	2.06 ^a^	0.44 ^b^	2.79 ^a^	0.453	0.002
Heptane	71	37.03 ^a^	8.96 ^b^	46.01 ^a^	6.667	0.001
Octane	85	121.25 ^a^	38.51 ^b^	156.27 ^a^	19.56	<0.001
2-Octene, (E)-	112	0.93 ^a^	0.36 ^b^	1.15 ^a^	0.150	0.002
Ethylbenzene (aromatic)	91	1.12 ^b^	3.09 ^a^	2.51 ^a^	0.338	0.001
Nonane	57	1.29 ^b^	1.06 ^b^	2.71 ^a^	0.295	0.001
Nonane, 5-methylene-	56	0.71	0.83	0.96	0.087	0.155
2,2,4,4- Tetramethyloctane	57	48.50	54.51	54.05	5.094	0.653
Undecane	57	2.13	2.75	2.36	0.253	0.225
Tridecane	57	0.32	0.33	0.33	0.054	0.991
Toluene (aromatic)	91	84.83	106.67	130.27	26.520	0.485
TOTAL HYDROCARBON CONTENT		300.17 ^ab^	217.51 ^b^	399.40 ^a^	30.242	0.001
2-Butanone	72	5.29	5.50	4.37	0.412	0.129
2-Pentanone	86	5.95	8.79	10.94	2.588	0.400
Acetoin	45	11.33	8.36	6.14	3.203	0.522
2-Hexanone	58	2.55	3.92	3.46	1.010	0.626
2-Heptanone	58	25.35	30.30	31.50	7.138	0.812
4-Hexen-3-one, 5-methyl	112	2.95 ^a^	1.05 ^b^	2.78 ^a^	0.284	0.001
2-Nonanone	58	4.21	4.96	6.45	1.395	0.517
TOTAL KETONE CONTENT		57.63	62.88	65.64	12.760	ns
Ethyl acetate	61	5.09	1.37	5.73	1.281	0.050
Propanoic acid, ethyl ester	57	1.45	0.84	1.54	0.396	0.397
Acetic acid, propyl ester	61	0.25	0.05	0.39	0.101	0.071
Propanoic acid, 2-methyl-, ethyl ester	116	1.41	0.68	1.31	0.331	0.251
Butanoic acid, ethyl ester	88	13.80	6.71	11.59	2.975	0.236
Butanoic acid, 2-methyl-, ethyl ester	102	7.45	3.92	9.11	2.358	0.293
Butanoic acid, 3-methyl-, ethyl ester	88	13.09	6.81	15.50	4.197	0.328
Acetic acid, 3-methylbutyl ester	70	0.77 ^b^	0.60 ^b^	2.68 ^a^	0.504	0.009
Acetic acid, 2-methylbutyl ester	70	0.17	0.07	1.18	0.347	0.052
Hexanoic acid, ethyl ester	88	35.28	16.38	41.32	10.74	0.241
Octanoic acid, ethyl ester	88	11.24	3.37	14.36	3.381	0.071
Decanoic acid, ethyl ester	88	2.08 ^ab^	0.58 ^b^	3.41 ^a^	0.766	0.041
TOTAL ESTER CONTENT		92.09	41.35	108.12	24.707	ns
Methanethiol	48	0.12 ^ab^	0.15 ^a^	0.07 ^b^	0.018	0.015
Trichloromethane	83	23.55 ^b^	16.08 ^b^	65.26 ^a^	11.990	0.012
Disulfide, dimethyl	79	10.84 ^b^	19.02 ^a^	8.98 ^b^	2.091	0.003
Butanoic acid	60	17.71 ^a^	8.56 ^b^	12.44 ^b^	1.357	<0.001
Furan, 2-pentyl-	81	12.31	13.96	14.71	1.919	0.667
Benzoic acid, m-hydroxyphenyl ester	105	1.11	0.64	0.76	0.220	0.305
Trisulfide, dimethyl	126	6.37 ^b^	10.80 ^a^	5.00 ^b^	1.250	0.005
TOTAL CONTENT OF OTHER COMPOUNDS		72.01	69.20	107.23	13.388	ns

VOCs—volatile organic compounds; ns—not significant; SIG—significance; SEM—standard error of the mean; M/Z—quantifier ion; SM—*semimembranosus*; BF—*biceps femoris*; ST—*semitendinosus*. Different lowercase letters (a, b, c) in the same column indicate significant differences between muscles (*p* < 0.05). ANOVA followed by a post hoc Tukey test.

## Data Availability

The original contributions presented in the study are included in the article, further inquiries can be directed to the corresponding author.

## References

[1] Leite A., Vasconcelos L., Ferreira I., Domínguez R., Pereira E., Rodrigues S., Lorenzo J.M., Teixeira A. (2023). Effect of the inclusion of olive cake in the diet on the physicochemical characteristics of dry-cured loin and dry-cured “cachaço” of Bísaro pig. Appl. Sci..

[2] Despacho nº16840/2005. 2ª Série. https://files.diariodarepublica.pt/2s/2005/08/149000000/1112411129.pdf.

[3] Fernández M., Ordoñez J.A., Cambero I., Santos C., Pin C., Hoz L. (2007). Fatty acids compositions of selected varieties of Spanish dry ham related to their nutritional implications. Food Chem..

[4] Domínguez R., Purriños L., Pérez-Santaescolástica C., Pateiro M., Barba J.F., Tomasevic I., Bastianello-Campagnol P.C., Lorenzo J.M. (2019). Caracterization of Volatile Compounds of dry-cured meat products using HS-SPME-GC/MS Techique. Food Anal. Methods.

[5] Ivanovic S., Nesic K., Pisinov B., Pavlovic I. (2016). The impact of diet on the quality of fresh meat and smoked ham in goat. Small Rumin. Res..

[6] Belloch C., Neef A., Salafia C., López-Diez J.J., Flores M. (2021). Microbiota and volatilome of dry-cured pork loins manufactured with paprika and reduced concentration of nitrite and nitrate. Food Res. Int..

[7] Martín-Mateos M.J., Amaro-Blanco G., Manzano R., Andrés A.I., Ramírez R. (2022). Efficacy of modified active packaging with oxygen scavengers for the preservation of sliced Iberian dry-cured shoulder. Food Sci. Technol. Int..

[8] Flores M., Durá M.A., Marco A., Toldrá F. (2004). Effect of *Debaryomyces* spp. on aroma formation and sensory quality of dry fermented sausages. Meat Sci..

[9] Bermúdez R., Franco D., Carballo J., Lorenzo J.M. (2014). Physicochemical changes during manufacture and final sensory characteristics of dry-cured Celta ham. Effect of muscle type. Food Control..

[10] Martínez-Onandi N., Rivas-Cañedo A., Ávila M., Garde S., Nuñez M., Picon A. (2017). Influence of physicochemical characteristics and high-pressure processing on the volatile fraction of Iberian dry cured ham. Meat Sci..

[11] Paié-Ribeiro J., Pinheiro V., Guedes C., Gomes M.J., Teixeira J., Leite A., Vasconcelos L., Teixeira A., Outor-Monteiro D. (2025). Exploring the Potential of Olive By-Products in Bísaro Pig Feed: Effects on the Chemical Compositions and Fatty Acid Profiles of Three Different Muscles. Foods.

[12] Pugliese C., Sirtori F., Škrlep M., Piasentier E., Calamai L., Franci O., Čandek-Potokar M. (2015). The effect of ripening time on the chemical, textural, volatile and sensorial traits of Bicep femoris and Semimembranosus muscles of the Slovenian drycured ham Kraški pršut. Meat Sci..

[13] Savić B., Škrlep M., Marušić Radovčić N., Petričević S., Čandek-Potokar M. (2025). Quality of Slovenian dry-cured ham from Krškopolje and hybrid pigs: Influence of skin trimming methods. Meat Sci..

[14] Ruiz-RamÍrez J., Arnau J., Serra X., Gou P. (2006). Effect of pH24, NaCl content and proteolysis index on the rela-tionship between water content and texture parameters in Biceps femoris and semimembranosus muscles in dry-cured ham. Meat Sci..

[15] Marušić Radovčić N., Poljanec I., Petričević S., Mora L., Medić H. (2021). Influence of Muscle Type on Physicochemical Parameters, Lipolysis, Proteolysis, and Volatile Compounds throughout the Processing of Smoked Dry-Cured Ham. Foods.

[16] Poljanec I., Marušić Radovčić N., Petričević S., Karolyi D., Listes E., Medić H. (2021). Proteolysis and protein oxidation throughout the smoked dry-cured ham process. Food Chem..

[17] Petričević S., Radovčić N.M., Lukić K., Listeš E., Medić H. (2018). Differentiation of dry-cured hams from dif-ferent processing methods by means of volatile compounds, physico-chemical and sensory analysis. Meat Sci..

[18] Council Regulation (EC) (2009). No 1099/2009 of 24 September 2009 on the protection of animals at the time of killing. Off. J. Eur. Communities.

[19] Álvarez-Rodríguez J., Teixeira A. (2019). Slaughter weight rather than sex affects carcass cuts and tissue composition of Bisaro pigs. Meat Sci..

[20] Specifications for “Presunto de Vinhais or Presunto Bísaro de Vinhais—PGI”. https://tradicional.dgadr.gov.pt/pt/cat/salsicharia-fumados-presuntos-e-paletas/1043-presunto-de-vinhais-igp-ou-presunto-bisaro-de-vinhais-igpentonces.

[21] (2008). Determinação do pH (Método de Referência); In Portuguese Norm–Meat and Meat Products.

[22] (2002). Determination of Moisture Content. Reference Method (ISO 1442:1197). In Portuguese Norm-Meat and Meat Products.

[23] (2002). Determination of Total Ashes. Reference Method (ISO 3496:1994). In Portuguese Norm-Meat and Meat Products.

[24] Folch J., Lees M., Stanley G.H.S. (1957). A simple method for isolation and purification of total lipids from animal tissues. J. Biol. Chem..

[25] (2002). Determination of Total Nitrogen Content. Determination of Total Nitrogen Content. Reference Method (ISO 937:1978). In Portuguese Norm-Meat and Meat Products.

[26] (1982). Meat and Meat Products—Determination of Chloride Content—Standard Method.

[27] (2002). Determination of Hydroxyproline Content. Reference Method.

[28] Hornsey H.C. (1956). The colour of cooked cured pork. I-Estimation of the nitric oxide-haem pigments. J. Sci. Food Agric..

[29] Cunniff P., AOAC International (1995). AOAC Official Methods of Analysis of AOAC International.

[30] Domínguez R., Borrajo P., Lorenzo J.M. (2015). The effect of cooking methods on nutritional value of foal meat. J. Food Compos. Anal..

[31] Teixeira A., Fernandes A., Pereira E. (2020). Fat content reduction and lipid profile improvement in Portuguese fermented sausages alheira. Heliyon.

[32] Ulbricht T.L.V., Southgate D.A.T. (1991). Coronary heart disease: Seven dietary factors. Lancet.

[33] Bermúdez R., Franco D., Carballo J., Lorenzo J.M. (2015). Influence of type of muscle on volatile compounds throughout the manufacture of Celta dry-cured ham. Food Sci. Technol. Int..

[34] de Bianchi T.L.C.F.P. (2013). Comparação de Processos Proteolíticos e Lipolíticos em Músculos de Presuntos Curados de uma População Suína Seleccionada de Acordo com Critérios Tecnológicos. Mestrado de Inovação e Qualidade na Produção Alimentar. Master’s Thesis.

[35] Domínguez R., Pateiro M., Gagaoua M., Barba F.J., Zhang W., Lorenzo J.M. (2019). A comprehensive review on lipid oxidation in meat and meat products. Antioxidants.

[36] Dominguez V.R. (2012). Influencia de la Alimentación Sobre los Ácidos Grasos, el Colesterol y el Retinol en Distintos Depósitos Grasos del Cerdo de Raza Celta. Ph.D. Thesis.

[37] Chen J., Liu H. (2020). Nutritional Indices for Assessing Fatty acids: A Mini-Review. Int. J. Mol. Sci..

[38] Department of Health and Social Care (1994). Nutritional aspects of cardiovascular disease. Report of the Cardiovascular Review Group Committee on Medical Aspects of Food Policy. Rep. Health Soc. Subj..

[39] Ramirez R., Cava R. (2007). Volatile profiles of dry-cured meat products from three different Iberian x Duroc genotypes. J. Agric. Chem..

[40] Jiang S., Xia D., Wang X., Zhu Y., Chen G., Liu Y. (2022). Analysis of aroma-active compounds in four Chinese dry-cured hams based on GC-O combined with AEDA and frequency detection methods. Food Sci. Technol..

[41] Sánchez-Peña C.M., Luna G., García-González D.L., Aparicio R. (2005). Characterization of French and Spanish dry-cured hams: Influence of the volatiles from the muscles and the subcutaneous fat quantified by SPME-GC. Meat Sci..

[42] Sirtori F., Dimauro C., Bozzi R., Aquilani C., Franci O., Pezzati A., Pugliese C. (2020). Evolution of volatile compounds and physical, chemical and sensory characteristics of Toscano PDO ham from fresh to dry-cured product. Eur. Food Res. Technol..

[43] Segura-Borrego M.P., Callejón R.M., Morales M.L. (2023). Iberian dry-cured ham sliced: Influence of vacuum packaging on volatile profile during chill-storage. Food Packag. Shelf Life.

[44] Górska E., Nowicka K., Jaworska D., Prybylski W., Tambor K. (2017). Relationship between sensory attributes and volatile compounds of polish dry-cured loin. Asian-Australas. J. Anim. Sci..

[45] Lorenzo J.M., Carballo J., Franco D. (2013). Effect of the inclusion of chestnut in the finishing diet on volatile compounds of dry-cured ham from celta pig breed. J. Integr. Agric..

[46] Nárváez-Rivas M., Vicario I.M., Alcade M.J., León-Camacho M. (2010). Volatile hydrocarbon profile of Iberian dry-cured hams. A possible tool for authentication of hams according to the fattening diet. Talanta.

[47] Wu W., Zhou Y., Wang G., Zhu R., Ge C., Liao G. (2020). Changes in the physicochemical properties and volatile flavor compounds of dry-cured Chinese Laowo ham during processing. J. Food Process Preserv..

[48] Gaspardo B., Procida G., Toso B., Stefanon B. (2008). Determination of volatile compounds in San Daniele ham using headspace GC-MS. Meat Sci..

[49] Sidira M., Kandylis P., Kanellaki M., Kourkoutas Y. (2015). Effect of immobilized Lactobacillus casei on volatile compounds of heat treated probiotic dry-fermented sausages. Food Chem..

[50] Zhang J., Wang L., Liu Y., Zhu J., Zhou G. (2006). Changes in the volatile flavour components of Jinhua ham during the traditional ageing process. Int. J. Food Sci. Techol..

[51] López-Salas L., Cea I., Borrás-Linares I., Emanuelli T., Robert P., Segura-Carretero A., Lozano-Sánchez J. (2021). Preliminary investigation of different drying systems to preserve hydroxytyrosol and its derivatives in olive oil filter cake pressurized liquid extracts. Foods.

[52] Bosse R., Wirth M., Becker T., Weiss J., Gibis M. (2017). Determination of volatile marker compounds in raw ham using headspace-trap gas chromatography. Food Chem..

